# In vitro antidiabetic and antioxidant activities of *Galega officinalis* extracts

**DOI:** 10.1002/fsn3.4326

**Published:** 2024-08-22

**Authors:** Shokoofeh Sukhtezari, Mohammad Ali Sahari, Mohsen Barzegar, Mohammad Hossain Azizi

**Affiliations:** ^1^ Department of Food Science and Technology, Faculty of Agriculture Tarbiat Modares University Tehran Iran

**Keywords:** antidiabetic activity, antioxidant activity, *Galega officinalis*, solvent extraction

## Abstract

The purpose of the present study was to determine the total phenolic, flavonoid, and galegine content and antioxidant activity, as well as the in vitro antidiabetic potential of different extracts of *Galega officinalis* using the solvent extraction method. The results demonstrated that the highest yield of extraction (28.05%) and galegine content (17.40 ± 0.04 μg/g of sample) was obtained using water as the solvent (*p* < .05). However, the highest total phenolic content (TPC) (138.35 ± 0.63 mg GAE per gram of dried GOEs) and total flavonoid content (TFC) (189.12 ± 1.47 mg catechin per gram of dried GOEs) were extracted using A90 (acetone–water, 90:10) solvent. A90 extract exhibited the highest inhibition of sucrase activity (91.42%) (*p* < .05). Also, the inhibitory activity of A90 against α‐amylase (59.96%), α‐glucosidase (54.3%), and maltase (62.73%) was significantly higher than that of A70 (acetone–water, 70:30) and E20 (ethanol–water, 20:80) (*p* < .05). According to antioxidant activity results, the highest ABTS^•+^ (360.5 ± 15.69 μmol Trolox eq per gram of dried GOEs), hydroxyl radical‐scavenging activity (3657.75 ± 21.56 μmol histidine eq per gram of dried GOEs), and FRAP assay (558.18 ± 20.26 μmol FeSO_4_ eq per gram of dried GOEs) were related to A90, while the best DPPH radical‐scavenging activity and metal‐chelating activity were related to A70 (302.66 ± 2.42 μmol Trolox equivalents per gram of dried GOEs) and E20 (36.5 ± 1.02 μmol EDTA eq per gram of dried GOEs), respectively. Taken together, A90 appears to be the best solvent to get *Galega officinalis* extract with the highest antioxidant and antidiabetic activity.

## Graphical abstract

This research aimed to determine the total phenolic, flavonoid, and galegine content, to evaluate the antioxidant (DPPH, ABTS+, and hydroxyl radical‐scavenging activity; ferric‐reducing antioxidant power; ferrous ion chelating) and enzyme inhibitory activities of *Galega officinalis* (GO) extracts obtained using solvents with different polarities. This is the first study about the inhibitory potential of GO against type 2 diabetic enzymes using in vitro models. α‐amylase, α‐glucosidase, sucrase, and maltase inhibitory activity of GOEs with a test concentration of 10 mg/mL. Different letters indicate significant differences among samples (*p* < .05). E: ethanol–water (20%, 50%, 70%, and 90% ethanol), A: acetone–water (20%, 50%, 70%, and 90% acetone), EA: ethanol–acetic acid (99:1), and AA: acetone–acetic acid (99:1).

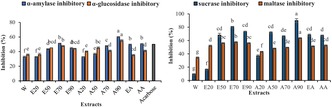

## 1 INTRODUCTION

The outbreak of diabetes is increasing throughout the world, and type 2 diabetes (T2D) poses the most threat among all types of diabetes, which is distinct by insulin resistance and damage to pancreatic beta cells (Hwang et al., 2015). Acarbose, gliclazide, and sitagliptin are some of the commercial drugs that are used to treat diabetes, but long‐term use of them leads to side effects (Gong et al., 2020). The estimation of the International Diabetes Federation (IDF) demonstrated that 536 million people were suffering from diabetes (diagnosed or undiagnosed) in 2021, which is expected to be 783 million people in 2045 (Ogurtsova et al., 2022). Hypertension, chronic hyperglycemia, and hyperlipidemia are important factors related to T2D due to insulin resistance or insulin deficiency (Kadan et al., 2016). These factors have been caused by a disturbance in the cellular redox balance and the induction of oxidative stress (Patel & Ghane, 2021; Tungmunnithum et al., 2018; Wang et al., 2019; Zhang, Xu, et al., 2020; Zhang, Li, et al., 2020). Antioxidants play a vital role in reducing oxidative stress by sustaining a balance between free radical generation and oxidative stress (Lim et al., 2019; Nawaz et al., 2020). Numerous studies have indicated that plant polyphenols prevent different oxidative stress‐induced diseases due to their antioxidant activities. (Ahmed et al., 2019; Familoni et al., 2019; Vergun et al., 2020). Secondary metabolites, such as polyphenols, are the most common group of compounds in plant materials and contain bindings between at least one hydroxyl group and one or more aromatic rings. Their antioxidant activity is induced by redox potential, which enables them to act as singlet metal chelators, oxygen quenchers, hydrogen donors, and reducing agents (Lalegani et al., 2018). The postprandial blood glucose level is an effective factor in the onset and aggravation of T2D (Chukwuma et al., 2020) that can be managed through the inhibition of carbohydrate‐digesting enzymes (Karimi et al., 2020; Wang et al., 2019; Xie et al., 2021). Phytochemical compounds such as polyphenols in plants can inhibit α‐amylase and α‐glucosidase activities and restrain T2D through binding to them (Sarteshnizi et al., 2021; Zhang et al., 2015).

*Galega officinalis* (GO) is a medicinal plant that has been traditionally utilized as a natural therapeutic for T2D in many countries for many years and can be cultivated as a garden plant (Bednarska et al., 2020; Hachkova et al., 2021). The main components of GO include phenols, flavonol triglycosides, alkaloids, terpenes, resins, steroids, and fatty acids (Khezri et al., 2022). Galegine is the main alkaloid of GO that has a chemical structure similar to metformin and led to the development of that as an antidiabetic drug (Pashazadeh et al., 2015). It was previously reported that the blood sugar‐reducing agent of GO is galegine, whereas new analyses showed that other phytochemical compounds, such as polyphenols, have hypoglycemia effects (Vergun et al., 2020).

The extraction of functional compounds from herbal resources, especially secondary metabolites, depends on multiple factors, including the type of solvents, solvent ratio, polarity, temperature, treatment time, organ composition, type of tissue, and age or stage of growth (Khezri et al., 2022). Solvent composition during extraction is one of the main effective parameters to determine the content and nature of secondary metabolites extracted from medicinal plants (Wang et al., 2019). For example, polar solvents are the preferred choice for extracting phenolic acids, flavonoids, and glycosides, while low‐polar or non‐polar solvents are more effective in extracting sterols, fatty acids, and fat‐soluble vitamins (Dirar et al., 2019). Due to the different polarity of solvents and the chemical nature of compounds, different phytochemicals can be extracted (Ullah et al., 2019). Additionally, combining two or more solvents can be effective in the extraction of all available antioxidants from plant materials (Nawaz et al., 2020). Utilization of water, ethanol, acetone, and acetic acid as extraction solvents resulted in the extraction of phenolic acids, flavonoids, alkaloids, glycosides, proteins, carbohydrates, and reducing sugars, which can be a useful approach to preparing extracts with high antioxidant and antidiabetic activities (Fernandes et al., 2019; Patel & Ghane, 2021; Wang et al., 2021).

However, according to our understanding, there is no research in the literature about the inhibition potential of GO against carbohydrate‐digesting enzymes (such as α‐glucosidase, sucrase, maltase, and α‐amylase). Moreover, there are limited studies on how solvent polarity affects the antidiabetic and antioxidant potential of GO extracts. Therefore, this research aimed to evaluate the antioxidant and enzyme inhibitory activity of GO extracts obtained using solvents with different polarities. Thus, this is the first study about the inhibitory potential of GO against T2D diabetic enzymes using in vitro models.

## 2 MATERIALS AND METHODS

### 2.1 Chemicals

*Galega officinalis* was prepared by the Zardband Co. (Tehran, Iran). gallic acid, Folin‐Ciocalteau reagent, 2,2‐diphenyl‐1‐picrylhydrazyl (DPPH), ferrozine, Trolox (6‐hydroxy‐2,5,7,8‐tetramethyl chroman‐2‐carboxylic acid), 2,2′‐azino‐bis (3‐ethylbenzothiazoline‐6‐sulfonic acid) diammonium salt (ABTS), potassium persulfate (Cat no. 7727‐21‐1), and 1,10‐phenanthroline (Cat no. 131377) were purchased from Sigma‐Aldrich Co. In addition, α‐amylase isolated from porcine pancreas (Cat no. A3176), PAHBAH (4‐hydroxybenzoic acid hydrazide), intestinal acetone powder from rats (Cat no. I1630), p‐nitrophenyl‐α‐D‐glucopyranoside (pNPG), sucrose, maltose, acarbose, soluble starch (Cat no. S9765), and glucose were obtained from Sigma‐Aldrich. Acetic acid, acetone, ethanol, 2,4,6‐tris‐(2‐pyridyl)‐s‐triazine (TPTZ), and l‐Histidine (Cat no. 104351) were purchased from the Merck Chemical Co. (Darmstadt, Germany). Galegine (Cat. no. AKOS000276789) was prepared by AKOS GmbH (Germany).

### 2.2 Methods

#### 2.2.1 Extraction

##### Preparation of *Galega officinalis* extracts (GOEs)

The plant material was air‐dried in the shade for one week, then ground and sieved through a No. 40 mesh sieve, and stored at −18 °C until use. To prepare the GOEs, various solvent systems were employed: water (W), ethanol–water at different ratios (20:80, 50:50, 70:30, and 90:10), acetone–water at different ratios (20:80, 50:50, 70:30, and 90:10), ethanol–acetic acid (99:1), and acetone–acetic acid (99:1). The extraction process involved stirring the mixture of solvent and plant powder at a speed of 100 rpm for 3 h. Then, the mixture was centrifuged at 14972 g for 5 min. The supernatant was filtered through a Whatman filter paper (No. 42) and concentrated under vacuum conditions at 40°C. Finally, the extracts were dried using a freeze‐drier at −57°C for 48 h (Labconco, USA). To calculate the percentage of extract yield, the following equation was used:

$$\text{Yield}\;\left(\%\right)=\left(W_{2}/W_{1}\right)\times 100$$

where *W*_2_ represents the weight of the dried GOE and *W*_1_ represents the weight of the dried GO (plant material).

##### Total phenolic content (TPC)

The total phenolic content of samples was measured following a procedure described by Lalegani et al. (2018). Briefly, 20 μL of GOE solution (1 mg/mL) was mixed with 1160 μL of deionized water. Then, 100 μL of Folin–Ciocalteu reagent was added to the mixture and kept for 5 min. Afterward, 300 μL of a 20% sodium carbonate solution was mixed with the samples and remained for 30 min at room temperature in the dark. The absorbance was read by a UV–Vis spectrophotometer (Cary 60, USA) at 765 nm. The TPC of GOEs was determined using a gallic acid (0–1000 ppm) standard curve and expressed as milligrams of gallic acid equivalents (mg GAE) per gram of dried GOEs.

##### Total flavonoids content (TFC)

The total flavonoid content of GOEs was determined according to Yuliani (2022) with slight modifications. Initially, 300 μL of GOE (1 mg/mL) and 150 μL of sodium nitrate (5%) were mixed and remained for 5 min. Then, 150 μL of 10% AlCl3 was added to the mixture and kept for 10 min. Afterwards, 1 mL of NaOH (1 M) was added to the solution and remained for 2 h at room temperature in the dark. The absorbance was monitored against blank at 352 nm, and TFC was calculated as milligrams of catechin equivalents (mg catechin) per gram of dried GOEs.

##### Galegine content (GC)

The galegine content of GOEs was analyzed using reversed‐phase high‐performance liquid chromatography (RP‐HPLC) with an Azura system from Knauer, Germany. The system was equipped with an LC pump (P 6.1 L), a UV–Vis detector (DAD 2.1 L), and an ODS3 reversed‐phase column (250 × 4.6 mm; Phenomenex, USA). The detection wavelength was 232 nm. The analysis was performed in an isocratic system, where the mobile phase consisted of potassium dihydrogen phosphate (0.05 M, pH = 3.5) and acetonitrile in a ratio of 70:30. The column oven temperature was set at 25°C. The flow rate was set at 1 mL/min, and the injection volume was 20 μL (Khezri et al., 2022). The equation of the standard curve used for the determination of galegine content was *y* = 9.5227*x* + 54.823 (*R* ^2^ = 0.9997).

#### 2.2.2 Antioxidant activity

##### DPPH radical‐scavenging activity (DPPH‐RSA)

The DPPH radical‐scavenging activity was determined by mixing GOE samples (1 mg/mL) with DPPH radical solution (0.1 mM) at a ratio of 100:900 and keeping the samples for 30 min in the dark. The absorbance of the samples was measured at 517 nm. The DPPH radical‐scavenging activity of GOEs was expressed as μmol Trolox equivalents per gram of dried GOEs (Noorolahi et al., 2020).

##### ABTS ^+^ radical‐scavenging activity (ABTS‐RSA)

ABTS^+^ radical‐scavenging potential was evaluated by Lim et al. (2019). Briefly, 1 mL of ABTS^•+^ (14 mM) was mixed with 1 mL of potassium persulfate (4.8 mM) solution and kept in the dark at 4°C for 14 h. Afterward, the mixture was diluted using distilled water to decrease the absorbance of the solution to 0.67 at 740 nm. To measure the ABTS^+^ radical‐scavenging activity, 20 μL of GOE was added to 980 μL of ABTS^+^ radical solution and remained in the dark for 10 min. The absorbance was read at 740 nm, and the results were reported as μmol Trolox equivalents per gram of dried GOEs.

##### Hydroxyl radical‐scavenging activity (HRSA)

The hydroxyl radical‐scavenging activity was determined by Karimi et al. (2020). Briefly, 400 μL of GOE (1 mg/mL) was added to 200 μL of phenanthroline solution (1.865 mM) and 200 μL of FeSO_4_ solution (1.865 mM). After 10 min in the dark, 200 μL H_2_O_2_ (0.03%, v/v) was added to the solution and remained for 1 h at 37°C. The absorbance was determined at 536 nm against the reagent blank. The results were calculated as:

$$\text{HRSA}\;\left(\%\right)=\left[\left(S–C\right)/\left(B–C\right)\right]\times 100$$

where *S*, *C*, and *B* show the absorbance of GOEs, negative control, and blank, respectively. The results were reported as μmol of histidine eq per gram of dried GOEs.

##### Ferric‐reducing antioxidant power (FRAP)

The ferric‐reducing antioxidant power evaluates the potential of antioxidants to reduce ferric (Fe^3+^) to ferrous (Fe^2+^). For preparing the FRAP solution, 5 mL of TPTZ (10 mM), 5 mL of FeCl_3_ (20 mM), and 50 mL of 0.3 M acetate buffer solution (pH = 3.5) were mixed. Then, 900 μL of FRAP solution was mixed with 100 μL of GOE solution (1 mg/mL) or FeSO_4_.7H_2_O (25, 50, 100, 200, 500, and 1000 mM) as the standard solution. The absorbance was read at 593 nm after keeping samples at 37°C for 30 min. FRAP was calculated using a standard curve of FeSO_4_ and reported as μmol of Fe^2+^ equivalents per gram of dried GOEs (Noorolahi et al., 2020).

##### Ferrous ion chelating activity (FICA)

The ferrous ion (Fe^2+^) chelating activity of GOEs was evaluated as follows. Briefly, 400 μL of GOE (1 mg/mL) was added to 2900 μL of distilled water and 500 μL of FeCl_2_ (2 mM) for 3 min at room temperature. Then, 200 μL of ferrozine (5 mM) was added and kept for 20 min. The absorbance was read at 562 nm. An EDTA standard curve (25–500 μM) was applied for calculating and reporting the results as μmol of EDTA equivalents per gram of dried GOEs (Karimi et al., 2021).

#### 2.2.3 Antidiabetic activity

##### α‐Amylase inhibition assay

The α‐amylase inhibition activity of GOEs was measured by Karimi et al. (2020). At first, a mixture of 100 μL of GOE (10 mg/mL) and α‐amylase solution (100 μL, 0.5 U/mL) was kept for 10 min at 37°C. Then, starch solution (100 μL, 0.5% (w/v)) was added to the mixture and, after 20 min, heated at 90°C for 10 min to stop the reaction. The mixture was centrifuged at 12,000 ***g*** for 5 min. At last, 25 μL of the supernatant was added to 1250 μL of PAHBAH and incubated at 70°C for 10 min. The absorbance was obtained at 410 nm, and the percentage of α‐amylase inhibitory activity was determined using the following equation:

$$\text{Inhibition of}\;\alpha –\text{amylase}\;\left(\%\right)=\left[1-\left(S-B\right)/C\right]\times 100$$

where *S*, *B*, and *C* show the absorbance of the sample, blank (the mixture of sample, enzyme, and buffer), and control (the mixture of enzyme, starch, and buffer), respectively. Acarbose (IC_50_ value) was used as the positive control.

##### α‐Glucosidase inhibition assay

The inhibition of rat intestinal α‐glucosidase was evaluated as follows: Initially, 0.2 g of rat intestinal acetone powder was added to 6 mL of phosphate buffer (0.02 M, pH = 6.9) containing NaCl (0.67 mM) and homogenized by an ultrasonic bath at 4°C (15 times, 30 second pulses). After centrifugation at 12,000 ***g*** for 30 min, the supernatant was diluted to the final concentration of 40 mU/mL. Then, 100 μL of GOE (10 mg/mL) was mixed with 200 μL of the enzyme solution and kept for 10 min at 37°C. Afterward, 100 μL of pNPG solution (5 mM) was added, and the mixtures were kept for 30 min. The absorbance was read at 405 nm. The inhibitory effect on α‐glucosidase was calculated using the following equation (Sarteshnizi et al., 2021):

$$\text{Inhibition of}\;\alpha –\text{glucosidase}\;\left(\%\right)=\left[\left(C-S\right)/C\right]\times 100$$

where *S* and *C* show the slope of the absorbance curve (3–18 min) for the control and samples, respectively. The positive control was acarbose.

##### Maltase and sucrase inhibitory assays

The inhibitory effect of GOE on sucrase and maltase activity was carried out as follows: the preparation of the enzyme solution was described in Section ‘α‐Glucosidase inhibition assay’ according to the (Lalegani et al., 2018) method. For the sucrose inhibition assay, 100 μL of enzyme solution and 100 μL of sucrose (40 mM), and for the maltase inhibition assay, 25 μL of enzyme solution and 175 μL of maltose (6 mM) were incubated with 50 μL of GOE (60 min for sucrase and 30 min for maltase) at 37°C. Then, the mixtures were heated at 100°C for 10 min to stop the reactions. The amount of glucose was analyzed by HPAEC‐PAD using the CarboPac PA1 analytical column (4 × 250 mm). Water and NaOH (0.5 mM) were used as the mobile phase with a flow rate of 0.8 mL/min. Sucrase and maltase inhibitions were calculated according to the following equation:

$$\text{Inhibition}\;\left(\%\right)=\left[\left(C-S\right)/C\right]\times 100$$

where *C* and *S* represent the glucose values of the control and samples containing GOEs, respectively. To compare the inhibitions, acarbose was considered a positive control.

#### 2.2.4 Statistical analysis

The analysis was performed using a one‐way analysis of variance (ANOVA), and the means were compared using the least significant difference (LSD) test (*p* < .05) by JMP statistical software. All measurements were done in triplicate, and the results were reported as the mean ± standard deviation. The Pearson's correlation test and the multivariate principal component analysis (PCA) were carried out using XLSTAT.

## 3 RESULTS AND DISCUSSION

### 3.1 Extraction

#### 3.1.1 Yield of extraction

Water, E20, E50, E70, E90, A20, A50, A70, A90, EA, and AA were used to extract GOEs. The results of GOE yield are shown in Table 1. The higher extraction yields were obtained using water and E20 (28.05 ± 0.62% and 26.42 ± 0.38%, respectively), and the lower yields belonged to AA and EA solvents (3.57 ± 0.45% and 2.1 ± 0.28%, respectively) (*p* < .05). In our study, more polar solvents had higher yields, which is consistent with the results of other studies (Boulfia et al., 2021; Tierney et al., 2013). Dirar et al. (2019) measured the effect of different solvents on the extraction of secondary metabolites from some medicinal plants grown in Sudan and noticed that increasing the concentration of water in hydroalcoholic solvents raised the extraction yield of *Dicoma tomentosa* and *Maerua pseudopetalosa*. Also, Nawaz et al. (2020) reported that extraction using water resulted in a high extraction yield of *Phaseolus vulgaris*. The extraction yields of water–ethanol solvents of *Trigonella foenum‐graecum* by increasing the percentage of ethanol decreased, as water and 96% ethanol had the highest and lowest TPC (Lohvina et al., 2021). It seems that extraction yield is significantly affected by the polarity of solvents, and high‐polar solvents show higher extraction yields. Farvin et al. (2019) reported that the higher yield of water extract might be related to the extraction of more polar and water‐soluble components such as proteins, peptides, and polysaccharides.

**TABLE 1:** Extraction yield, TPC, TFC, and GC of GOEs.

Samples	Extract yield (%)	TPC (mg GAL/g of GOEs)	TFC (mg cat/g of GOEs)	GC (μg/g of GOEs)
W	28.05 ± 0.62^a^	81.05 ± 0.42^c^	61.46 ± 0.65^g^	17.40 ± 0.04^a^
E 20	26.42 ± 0.38^a^	86.01 ± 1.08^c^	101.95 ± 0.78^de^	14.67 ± 0.01^b^
E 50	23.98 ± 0.51^b^	101 ± 1.01^bc^	129.95 ± 3.89^bc^	10.39 ± 0.01^de^
E 70	18.38 ± 0.52^c^	123.5 ± 3.11^ab^	139.72 ± 0.36^b^	10.25 ± 0.08^e^
E 90	16.22 ± 0.67^d^	100.2 ± 2.71^bc^	108.74 ± 0.95^de^	10.08 ± 0.03^de^
A 20	23.69 ± 0.64^b^	98.6 ± 1.83^bc^	90.13 ± 3.83^ef^	14.63 ± 0.01^b^
A 50	22.33 ± 0.66^b^	93.45 ± 1.13^bc^	118.08 ± 1.59^cd^	11.52 ± 0.01^c^
A 70	15.95 ± 0.42^d^	106.45 ± 0.68^bc^	142.43 ± 1.32^b^	10.79 ± 0.01^d^
A 90	10.1 ± 0.28^e^	138.35 ± 0.63^a^	189.12 ± 1.47^a^	10.51 ± 0.01^de^
EA	3.57 ± 0.45^f^	104.65 ± 1.32^bc^	98.92 ± 0.42^de^	7.12 ± 0.01^g^
AA	2.1 ± 0.28^f^	90.5 ± 0.35^c^	74.26 ± 0.46^fg^	7.73 ± 0.01^f^

*Note*: Values are expressed as mean ± SD, *n* = 3.

Abbreviations: E: ethanol‐water (20, 50, 70, and 90% ethanol), A: acetone‐water (20, 50, 70, and 90% acetone), EA: ethanol‐acetic acid (99:1), AA: acetone‐acetic acid (99:1). Values with different letters within each time have significantly different mean values (*p* < 0.05). There were three independent replications for each treatment and the results are shown as mean ± standard deviation.

#### 3.1.2 Total phenolic content (TPC)

The results presented in Table 1 show that the total phenolic content (TPC) of the extracts ranged from 81.05 ± 0.42 to 138.35 ± 6.29 mg GAE per gram of dried GOEs. The highest TPC was found in the A90, followed by the E70 and A70 extracts, while the lowest content was observed in the water, E20, and AA extracts. These findings are consistent with previous studies that have shown the choice of solvent has a significant impact on the amount of TPC extracted from plants. Lohvina et al. (2021) demonstrated that a 70% ethanol extract of *Trigonella foenum‐graecum* resulted in a higher TPC than 96, 50, and 30% ethanol. Hachkova et al. (2021) reported that the type and ratio of solvents significantly affected the content of TPC extracts from Yacon leaves. Additionally, the extraction of the phenolic content of *Phaseolus vulgaris* seeds by non‐polar solvents is higher than polar ones (Nawaz et al., 2020). Lapornik et al. (2005) showed that water exploits the lowest TPC compared to 70% ethanol for black currant, red currant, and grape marc. Patel and Ghane (2021) reported that the lowest and highest TPC of *Luffa echinata* leaves belonged to water and acetone as solvent extraction, respectively. Different plant materials need different types of solvents for the extraction of polyphenol compounds (Wang et al., 2009). In our study, these observations suggest that less polar solvents might be more suitable for extracting polyphenols from GOEs.

#### 3.1.3 Total flavonoid content (TFC)

Flavonoids are a class of polyphenols with considerable biological activities that rely on the substitution pattern of aromatic rings containing at least one hydroxyl group (Tungmunnithum et al., 2018). The total flavonoid content of GOEs is shown in Table 1. The TFC of the extracts was in the range of 61.46 ± 0.65 to 189.12 ± 1.47 mg of catechin per gram of dried GOEs. The highest flavonoid content of GOEs was related to A90, followed by A70 extracts, whereas water extract had the lowest TFC. Dirar et al. (2019) reported that aqueous‐organic and organic solvents with lower polarity can extract the higher‐content flavonoid compounds of *Guiera senegalensis*. Ma et al. (2021) investigated the TFC of *Huangshan gongju* extracts, and the results showed that E70 had the highest TFC in the series of ethanolic extracts while in the series of acetone, A90 possessed the maximum flavonoid content. Dorta et al. (2012) showed that the lowest TFC of mango peel belonged to water extract, while aqueous‐organic extracts had the highest content of flavonoids. Boulfia et al. (2021) indicated that the aqueous extract from *Leopoldia comosa* had lower contents of flavonoid compounds than the organic extracts. Goulas and Georgiou (2019) found that acetone–water mixtures were better solvents for the extraction of flavonoids from carob fruit; in contrast, extracts of pure solvents contain lower amounts of flavonoids.

#### 3.1.4 Galegine content

**FIGURE 1:**
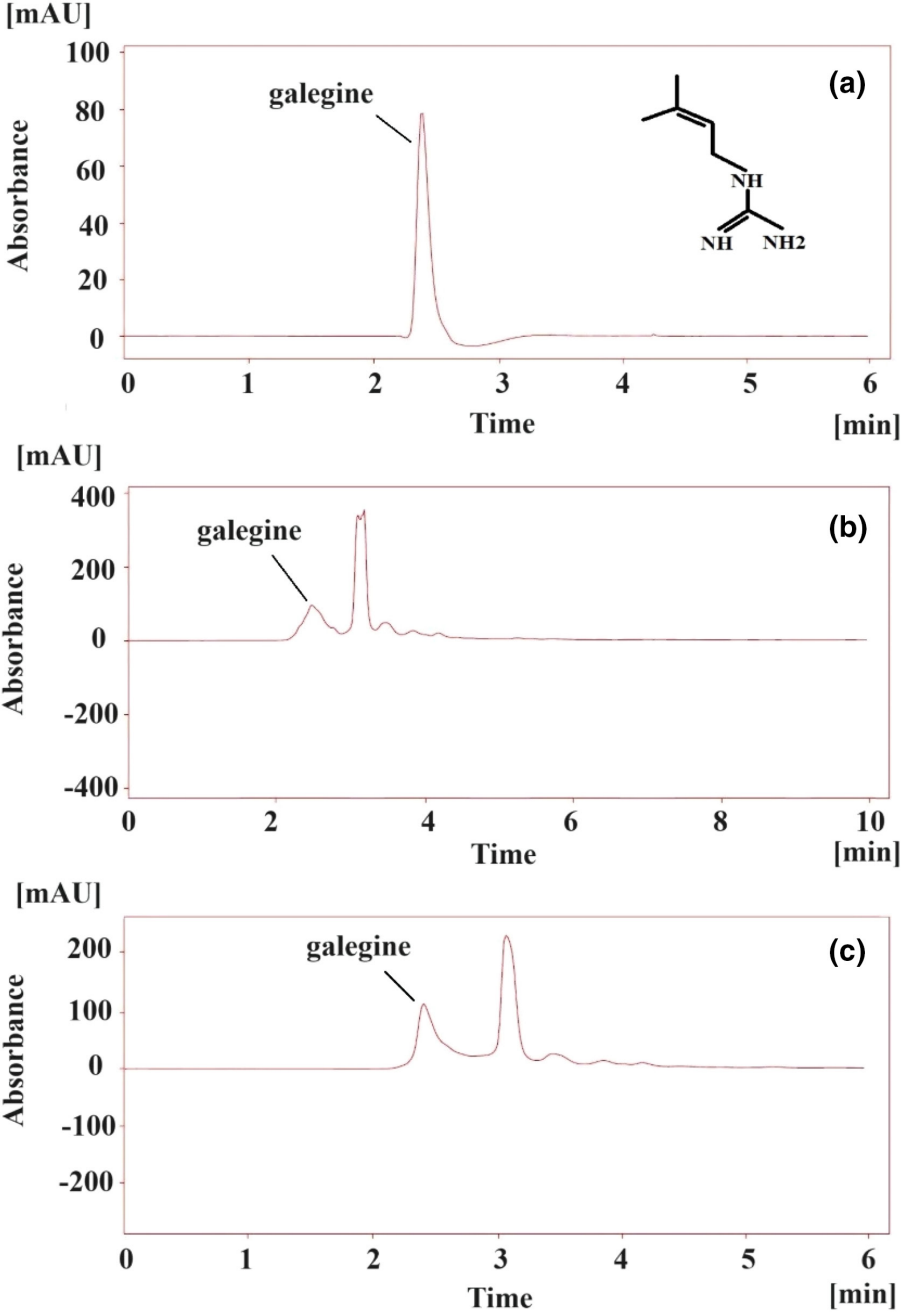
Chromatograms of galegine analysis. (a) HPLC chromatogram of standard galegine showing a single peak of galegine, (b) HPLC chromatogram of GOE showing the presence of galegine, and (c) HPLC chromatogram of GOE spiked with standard galegine.

Galegine is an alkaloid of *Galega officinalis* that has the potential to treat diabetes through its role in developing the production of antidiabetic drugs such as metformin, so improving the extraction of that from GO was investigated in previous studies (Vergun et al., 2020). We determined the impact of different solvents on obtaining the galegine of GO. Furthermore, RP‐HPLC using a UV detector has been used as a reliable procedure to detect alkaloids (McCalley, 2002), such as galegine (Khezri et al., 2022). Thus, the galegine content of different extracts was measured. The retention time of galegine (approximately 2.27 min) was detected by injection of galegine standard, and the results are indicated in Figure 1. The results of the galegine content obtained using different solvents are shown in Table 1. The galegine contents of aqueous and aqueous‐organic extracts were significantly different (*p* < .05). The highest value of this parameter was found in the water extract (17.70 ± 0.07 mg per gram of dried GOEs), while the lowest value was in the AA extract (7.73 ± 0.01 mg per gram of dried GOEs) (*p* < .05). Alkaloids are secondary metabolites containing nitrogen atoms that, due to their chemical structure and solubility, have different yield extraction in different solvents (Adejoke et al., 2019), and in our samples, water was the best solvent to extract the galegine from *Galega officinalis*.

### 3.2 Antioxidant activity

#### 3.2.1 DPPH radical‐scavenging activity (DPPH‐RSA)

**FIGURE 2:**
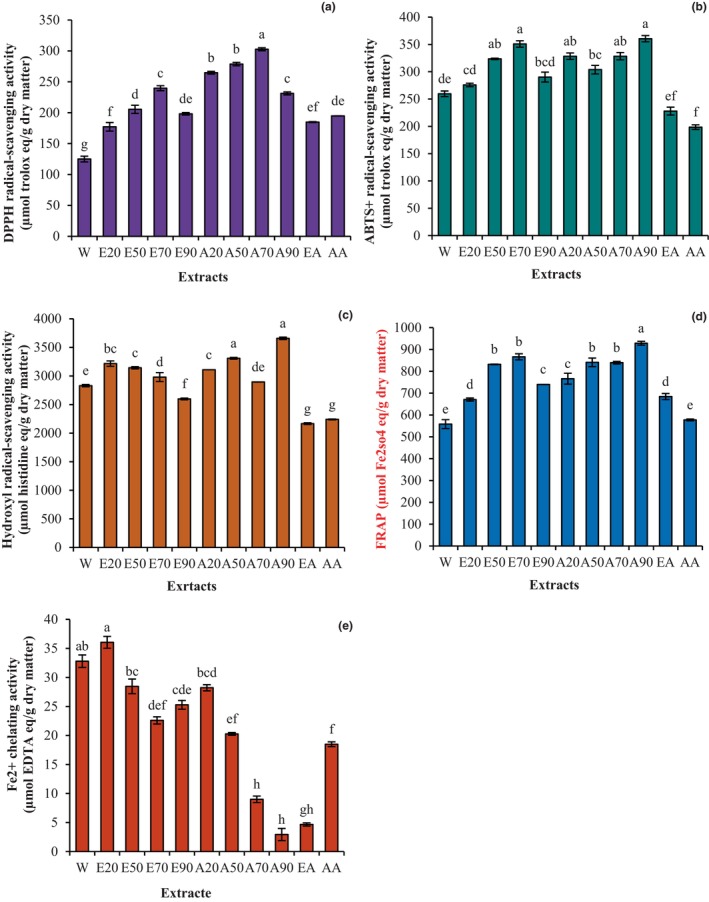
Antioxidant activity of GOEs: (a) DPPH, (b) ABTS^+^, (c) hydroxyl radical scavenging, (d) FRAP, and (e) Fe^2+^ chelating activity of GOEs. The test concentration was 1 mg/mL. Data marked with different letters are significantly different (*p* < .05). A, Acetone–water (20%, 50%, 70%, and 90% acetone); E, Ethanol–water (20%, 50%, 70%, and 90% ethanol); AA, Acetone–acetic acid (99:1); EA, Ethanol–acetic acid (99:1).

Antioxidant activity is influenced by diverse factors, and a single antioxidant property model cannot comprehensively represent the antioxidant capacity of all samples. Hence, various antioxidant activity assays are employed to capture the multifaceted mechanisms of antioxidant action. Figure 2a shows the DPPH‐RSA of GOEs. The strongest DPPH‐RSA was observed in A70, with 302.66 ± 2.42 μmol Trolox equivalents per gram of dried GOEs (*p* < .05). This was followed by A50, A20, and E70 (278.61 ± 2.75, 264.66 ± 2.29, and 239.64 ± 4.0 μmol Trolox equivalents per gram of dried GOEs, respectively). The aqueous extract had a lower radical‐scavenging capacity than other solvents (125 ± 4.65 μmol Trolox equivalents per gram of dried GOEs). The polarity of solvents affects the efficiency of extracting phenolic and antioxidant compounds (Wang et al., 2019). Srivastava et al. (2020) indicated that among three solvents (acetone, ethanol, and methanol) at different concentrations, 70% acetone was the best solvent for DPPH‐RSA of *Feronia limonia* fruit. Several studies have shown that water extracts have a lower DPPH‐RSA compared to aqueous–alcoholic extracts. For example, Wang et al. (2009) reported that 70% acetone extract from several icelandic seaweeds has higher antioxidant activity than water extract from them. Similarly, Lapornik et al. (2005) measured the DPPH‐RSA of black currant, red currant, and grape marc with different solvents and found that 70% ethanol solvent had stronger antioxidant capacity than water. Also, the water extract of mango seed was a lower DPPH‐RSA scavenger than water–alcoholic extracts (Dorta et al., 2012).

#### 3.2.2 ABTS ^+^ radical‐scavenging activity

The ABTS^+^‐RSA of GOEs is shown in Figure 2b. The A90 and E70 extracts had the highest ABTS^+^‐RSA (360.5 ± 15.69 and 350.8 ± 16.68 μmol Trolox equivalents per gram of dried GOEs, respectively), followed by the A70 and A20 extracts. However, the lowest ABTS^+^‐RSA was obtained by AA solvent (198.5 ± 14.28 μmol Trolox equivalents per gram of dried GOEs (*p* < .05)). These results exhibited that although water is efficient in extracting ABTS‐reactive compounds, it is not the most effective solvent on its own, and a combination of water and alcohols is proven to be a more effective strategy. Ullah et al. (2019) indicated that to compare the ABTS^+^‐RSA of *Arachis hypogaea* root extracts, 70% ethanol, followed by 70% acetone, were the strongest solvents in contrast to water. Ma et al. (2021) studied the effect of different solvent extractions on the antioxidant activity of *Huangshan gongju* extracts and reported that 70% ethanol had the maximum antioxidant activity; in contrast, 90% acetone had a low antioxidant capacity, and water was the weaker extract. The result of ABTS^+^‐RSA of *Kaempferia rotunda* showed that water–acetone and water–ethanol extracts had a stronger scavenging potential than absolute acetone, ethanol, and water extract (Seno et al., 2023).

#### 3.2.3 Hydroxyl radical‐scavenging activity (HRSA)

The inhibition of two mechanisms that affect hydroxyl radical production, including binding the hydrogen peroxide to metal ions and one‐electron transfer to the free radicals, has a vital role in hydroxyl radical‐scavenging activity (Lim et al., 2019). The HRSA of GOEs is shown in Figure 2c. Similar to DPPH and ABTS^+^ radical‐scavenging activity results, the A90 extract with 3657.75 ± 21.56 μmol histidine equivalents per gram of dried GOEs showed the highest OH^•^ scavenging capacity, followed by the A50 extract with 3309.5 ± 14.84 μmol histidine equivalents per gram of dried GOEs (*p* < .05). However, EA and AA extracts had the lowest hydroxyl‐scavenging activity (2166 ± 13.43 and 2239.25 ± 6.71 μmol histidine equivalents per gram of dried GOEs, respectively). Zhang, Xu, et al. (2020), Zhang, Li, et al. (2020) showed that the acetone extract of *Erigeron annuus* flower exhibited a strong ability to scavenge hydroxyl radicals, followed by ethanol and water extracts. Fasakin et al. (2011) investigated different extracts of *Vernonia amygdalina* and *Gongronema latifolium* and reported that 80% acetone was the best solvent to scavenge radical hydroxyl. Wang et al. (2021) showed that the highest HRSA of *Areca catechu* aqueous‐organic extracts was found in 50% methanol extract, followed by 50% acetone and ethanol. The HRSA of various extracts is likely related to the differences in the composition and content of antioxidants in the extracts (Zhang, Xu, et al., 2020; Zhang, Li, et al., 2020).

#### 3.2.4 Ferric‐reducing antioxidant power (FRAP)

The FRAP method can detect secondary metabolites (redox‐active organic compounds) via the reduction of ferric ions to ferrous ions (Fasakin et al., 2011). The FRAP value of GOEs is indicated in Figure 2d. The lowest and highest FRAP were found for water extract and A90 extract (558.18 ± 20.26 and 928.44 ± 8.74 μmol FeSO_4_ equivalents per gram of dried GOEs), respectively (*p* < .05). Boulfia et al. (2021) found that aqueous extracts of *Leopoldia comosa* had a lower reducing power compared to organic extracts. The FRAP of *Salacia chinensis* extracts showed that 50% acetone and 50% ethanol extracts were the stronger scavengers than absolute acetone, ethanol, and water extracts (Ngo et al., 2017). Familoni et al. (2019) investigated the FRAP of water and organic extracts of *Acalypha godseffiana* leaves and reported that acetone was the more efficient solvent. In contrast, the results of Wang et al. (2019) demonstrated that as the water containment of solvents was decreased the antioxidant activity of Pyracantha fortuneana fruit extracts was increased. Do et al. (2014) reported that 100% ethanol extract had the highest reducing power of *Limnophila aromatica*, followed by 100% acetone extract, 100% methanol extract, and aqueous‐organic extracts, while water extract had the lowest reducing power. Thus, the combination of organic solvents and water is an effective procedure to extract antioxidant compounds.

#### 3.2.5 Ferrous ion chelating activity (FICA)

The Fe^2+^ chelating activity of different extracts is shown in Figure 2e. The ferrous ion chelating activity of extracts ranged from 2.93 ± 104 to 36.5 ± 1.02 μmol EDTA equivalents per gram of dried GOEs. Our findings revealed that E20 extract had significantly higher ferrous ion chelating activity, followed by water extract (36.5 ± 1.02 and 32.8 ± 2.08 μmol EDTA equivalents per gram of dried GOEs, respectively) (*p* < .05). Also, A90 showed the lowest FICA (2.93 ± 1.04 μmol EDTA equivalents per gram of dried GOEs). Wang et al. (2009) reported that the water extract of *Icelandic seaweeds* was a stronger Fe^2+^ chelator than the water–acetone solvent. Srivastava et al. (2020) compared the effect of organic solvent content on the antioxidant activity of *Feronia limonia* and observed that acetone 90% chelated Fe^2+^ lower than acetone 70% and 50%. The water extract of *Secamone afzelii* leaves showed the highest FICA, followed by 50%, 80%, and 100% water–methanol extracts (Sinan et al., 2023). Metal‐chelating activity did not follow the same trends observed for DPPH, ABTS^•+^, HRSA, and FRAP results, and there was also no correlation between FICA, TPC, and TFC (Belkhiri et al., 2017; Mamache et al., 2020). Thus, other antioxidant compounds such as amino acids, peptides, proteins, or polysaccharides might be better chelators of metal ions (Farvin et al., 2019). Marinaccio et al. (2023) reported the antioxidant potential of an extracted peptide from *spinach Rubisco*. A number of researchers have reported that compounds containing two or more ‐NR2 groups (such as galegine) can chelate the metal ions (Kolak et al., 2006; Shaheen et al., 2005).

### 3.3 Antidiabetic activity

#### 3.3.1 α‐Amylase inhibition assay

**FIGURE 3:**
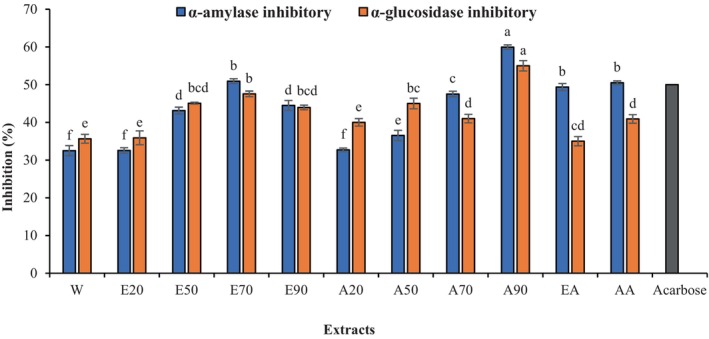
α‐amylase and α‐glucosidase inhibitory activity of GEO. The test concentration was 10 mg/mL. Acarbose was used as a positive control (IC50 = 110 μg/mL). Different letters indicate significant differences among the samples (*p* < .05). A, Acetone–water (20%, 50%, 70%, 90% acetone); E, Ethanol–water (20%, 50%, 70%, 90% ethanol); AA, Acetone–acetic acid (99:1); EA, Ethanol–acetic acid (99:1).

One of the most important reasons for postprandial hyperglycemia is increasing the level of blood glucose in the body. Amylases are a group of carbohydrate‐digesting enzymes that participate in catalyzing the breaking down of dietary polysaccharides and oligosaccharides to glucose (Patel & Ghane, 2021). Inhibiting these enzymes can delay the production of glucose and decrease postprandial hyperglycemia (Irondi et al., 2017). The results of α‐amylase inhibitory activity are indicated in Figure 3. Significantly higher inhibition potential against α‐amylase was observed in A90 (59.96%), followed by E70, EA, and AA extracts (*p* < .05). Water, E20, and A20 extracts displayed the lowest inhibition of α‐amylase. The previous studies show that plant materials such as pistachio green hull (Lalegani et al., 2018), nigella sativa seeds (Varghese & Mehrotra, 2020), blackberry juice (Buljeta et al., 2022), and *Cosmos caudatus* (Safitri et al., 2022) inhibited α‐amylase. Boulfia et al. (2021) found that *Juglans regia* bark acetone extract had the highest α‐amylase inhibition activity, followed by ethanol and water extracts. Hwang et al. (2015) exhibited that a 70% acetone extract of *Sargassum hemiphyllum* could inhibit α‐amylase in contrast to water and 95% ethanol, which failed to inhibit it. Also, 80% and 100% aqueous‐methanolic extracts of *Secamone afzelii* leaves demonstrated significantly higher α‐amylase inhibitory activity than the water extract, with an approximately 5‐fold difference (Sinan et al., 2023). The proposed mechanisms of enzymatic inhibition are the formation of covalent bonds or hydrogen bonds between polyphenols and the active site of enzymes, as well as the changing of starch microstructure by occurring non‐covalent binding between polyphenols and starch (Chai et al., 2013; Ćorković et al., 2022; Sun et al., 2020).

#### 3.3.2 α‐Glucosidase inhibition assay

Alpha‐glucosidase is an enzyme that hydrolyzes the sugars (aryl or alkyl‐glucosides, disaccharides, or oligosaccharides) and releases α‐glucose from the non‐reducing ends (Ćorković et al., 2022). The interaction of polyphenols with these enzymes can inhibit their activities (Gong et al., 2020). The inhibitory effect of GOEs on α‐glucosidase is indicated in Figure 3. A90 and water extracts showed the maximum (54.3%) and minimum (35.65) α‐glucosidase inhibitory activities, respectively (*p* < .05). It can be observed that the A90 extract with lower polarity exhibited the highest α‐glucosidase inhibition potential, which is in agreement with the result of the previous research (Wang et al., 2021). Sunagar and Sreerama (2023) reported that 80% acetone and water had stronger and weaker α‐glucosidase inhibitory activities for *Urochloa ramosa*, respectively. On the contrary, Dirar et al. (2019) found that water is a better solvent for the extraction of α‐glucosidase inhibitor metabolites from *Guiera senegalensis* than aqueous‐ethanol. Wang et al. (2019) showed that 50% acetone extract with the highest TPC and TFC was the strongest α‐glucosidase inhibitor for *Pyracantha fortuneana* fruit. It is important to note that flavonoids can bind to enzymes in two ways, including competitive inhibition, where they bind directly to the active site and block the substrate from binding, or non‐competitive inhibition, where they bind to a site near the active site and change the shape of the enzyme so that the substrate cannot bind (Zhu et al., 2020). Previous reports have indicated that the type of bioactive materials affected the amount of inhibitory activity of these compounds on carbohydrate digesting‐enzymes (Abdelhady et al., 2016; Fettach et al., 2019; Wang et al., 2019).

#### 3.3.3 Maltase and sucrase inhibitory assay

**FIGURE 4:**
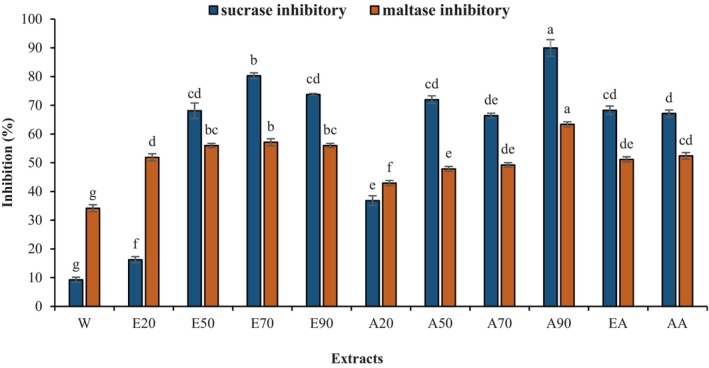
Sucrase and maltase inhibitory activity of GOEs with a test concentration of 10 mg/mL. Different letters indicate significant differences among samples (*p* < .05). A, Acetone–water (20%, 50%, 70%, and 90% acetone); AA, Acetone–acetic acid (99:1); E, Ethanol–water (20%, 50%, 70%, and 90% ethanol); EA, Ethanol–acetic acid (99:1).

Alpha‐glucosidase is a hydrolyzing enzyme involved in the amylolytic process of animals, plants, and microorganisms, and due to the differences in type of substrate, it is classified into three groups of enzymes. Group I breaks down heterogeneous substrates such as sucrose, whereas groups II and III break down homogeneous substrates such as maltose (Auiewiriyanukul et al., 2018; Ćorković et al., 2022). Since hydrolyzing these substrates to their constituent monomers raises the blood sugar level, inhibition of digestive enzyme activity seems an efficient approach to the limitation of glucose production (Nijpels et al., 2008), and bioactive compounds are potential inhibitors against carbohydrate‐hydrolyzing enzymes (Williamson, 2013; Zhang et al., 2015). The validation of hydrolysis needs to identify at least one of the released sugars, and HPLC is the best method to measure the values of sugars (Lalegani et al., 2018). Thus, we assessed the inhibitory effects in vitro of GOEs on maltase and sucrase activities. The results suggested that all of the extracts could significantly inhibit the evaluated enzymes. The inhibition rate for maltase ranged from 33 to 63%; however, the sucrase inhibition rate was in the range of 19–91%. Moreover, as shown in Figure 4, A90 exhibited significantly higher maltase and sucrose inhibitory activity compared to water extract (62.73% vs. 33.30% and 91.42% vs. 8.24%) (*p* < .05). Hwang et al. (2015) showed that the 70% acetone extract of *Sargassum hemiphyllum* was the strongest sucrase and maltase inhibitor due to the highest polyphenol content. Toma et al. (2014) reported that the sucrase inhibition of a 70% ethanol extract from *Moringa stenopetala* leaves was stronger than maltase and α‐amylase inhibition.

### 3.4 Correlation between bioactive content, antioxidant, and antidiabetic activities assays

**FIGURE 5:**
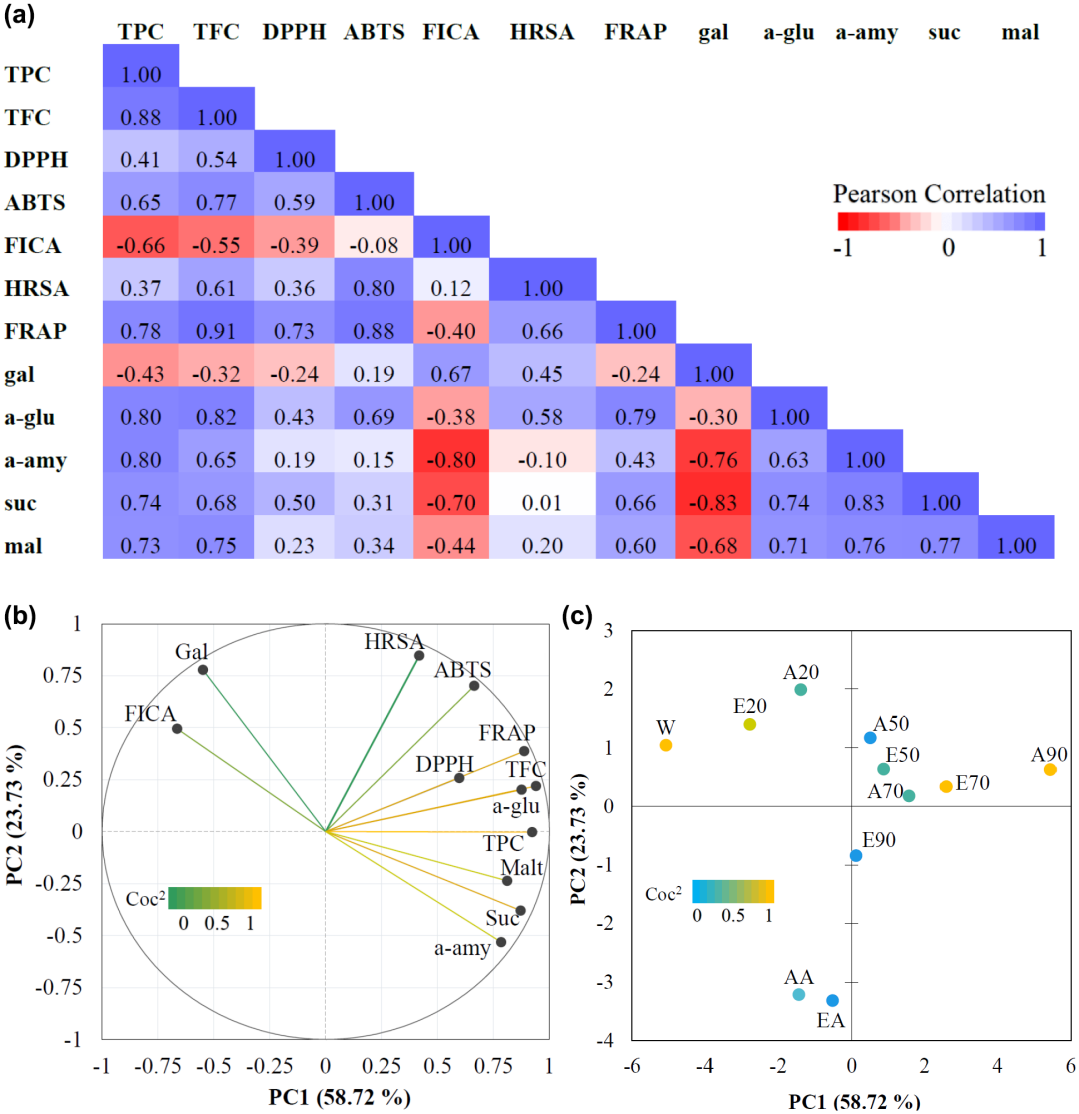
Pearson’s correlation test (a), PCA factor map (b), and score plot (c) of total phenolic content (TPC), total flavonoid content (TFC), and galegine content (gal), DPPH radical scavenging activity, ABTS+ radical scavenging activity, radical hydroxyl scavenging activity (HRSA), FRAP, and ferric ion chelating activity (FICA), α‐glucosidase (a‐glu), α‐amylase (a‐amy), sucrase (suc), and maltase (mal) inhibitory activity for GOEs (E: ethanol‐water (20, 50, 70, and 90% ethanol), A: acetone‐water (20, 50, 70, and 90% acetone), EA: ethanol‐acetic acid (99:1), AA: acetone‐acetic acid (99:1).

The correlation coefficient between TPC, TFC, GC, antioxidant activity (DPPH, ABTS, HRSA, FRAP, and FICA), and enzyme inhibitory activity (α‐glucosidase, α‐amylase, sucrase, and maltase inhibitory activity) of different extracts is indicated in Figure 5a. Principal component analysis (PCA) was also carried out to assess the relationship between the phytochemical, antioxidant, and antidiabetic activity of extracts. The most total variance of the data (82.45%) was observed in the first two principal components (PC1:58.72% and PC2:23.73%) (Figure 5b,c). The square cosine (cos (Adejoke et al., 2019)) values and color gradient were utilized to visualize the effectiveness of the representation in PCA and higher values resulted in better representation of variables. A narrow angle between variables on a factor plot reflects a positive correlation while a wide angle implies a negative correlation. The distance of a variable from the origin represents its quality, and variables with a larger distance are represented better on the factor map. As shown in the factor map, TPC, TFC, FRAP, α‐glucosidase, sucrase, maltase, and α‐amylase positioned on the right along PC1 as opposed to galegine and FICI (Figure 5b) which indicated the positive correlation between the first group of variables and negative correlation of them with the second group. In consistency with our results, Stefanucci et al. (2020) reported the strong correlation between bioactive compounds content and antioxidant and enzyme inhibitory potential of *Viscum album* extracts. The positive correlation between TPC and TFC with the antioxidant and antidiabetic potential of extracts suggests that phenols and flavonoids are important bioactive agents, which can scavenge free radicals and reduce ions (Jablonsky et al., 2020; Srivastava et al., 2020; Wang et al., 2019; Zhang, Xu, et al., 2020; Zhang, Li, et al., 2020) and inhibit carbohydrate‐digesting enzymes (Dirar et al., 2019; Hwang et al., 2015). On the other hand, a notable positive correlation between FICA and galegine (0.67) may be due to the galegine structure (Farvin et al., 2019; Jang et al., 2009).

## 4 CONCLUSIONS

In conclusion, the present research results revealed that *Galega officinalis* contains phytochemical materials with antidiabetic and antioxidant activities. Furthermore, the amount and type of these compounds are affected by the polarity of solvents. In our study, A90 (acetone 90%) extract in comparison with other extracts, demonstrated the highest bioactive content (total phenolic and flavonoids), antioxidant activity (ABTS^+^ radical scavenging activity, hydroxyl radical scavenging activity, and FRAP), also carbohydrate‐digesting enzyme (α‐glucosidase, α‐amylase, sucrase, and maltase inhibitory activity). In comparison to aqueous–acetone and aqueous–ethanolic extracts, water extract showed a higher extraction yield and galegine content. The metal‐chelating activity of E20 was remarkably high, despite its low polyphenol content, which can result from the presence of other compounds such as proteins, peptides, amino acids, or other polysaccharides. Therefore, A90 was suggested as the best solvent in terms of oxidation‐preventing agents and enzyme inhibitors. In general, *Galega officinalis* has remarkable potential to be an antidiabetic plant to control T2D, as both polyphenols and galegine can play a vital role in decreasing the fasting blood glucose level. This research demonstrated the antidiabetic and antioxidant potential of *Galega officinalis* extracts, which is beneficial for developing antihyperglycemic agents. In vivo hypoglycemic activity assays should be performed to further investigate the antidiabetic capacity of GOEs.

## AUTHOR CONTRIBUTIONS

**Mohammad Ali Sahari:** Conceptualization (equal); data curation (equal); formal analysis (equal); funding acquisition (equal); investigation (equal); project administration (equal); resources (equal); validation (equal); writing – original draft (equal); writing – review and editing (equal). **Shokoofeh Sukhtezari:** Conceptualization (equal); formal analysis (equal); investigation (equal); methodology (equal); software (equal); visualization (equal); writing – original draft (equal). **Mohsen Barzegar:** Methodology (equal); validation (equal); visualization (equal). **Mohammad Hossein Azizi:** Software (equal); supervision (equal).

## CONFLICT OF INTEREST STATEMENT

The authors note that there is no known conflict of interest in this publication.

## Data Availability

The authors do not have permission to share data.

## References

[fsn34326-bib-0001] Abdelhady, M. I. , Shaheen, U. , Bader, A. , & Youns, M. A. (2016). A new sucrase enzyme inhibitor from *Azadirachta indica* . Pharmacognosy Magazine, 12(Suppl 3), S293–S296. 27563214 10.4103/0973-1296.185705 PMC4971946

[fsn34326-bib-0002] Adejoke, H. T. , Louis, H. , Amusan, O. O. , & Apebende, G. (2019). A review on classes, extraction, purification and pharmaceutical importance of plants alkaloid. Journal of Medicinal and Chemical Sciences, 2, 130–139.

[fsn34326-bib-0003] Ahmed, A. F. , Attia, F. A. , Liu, Z. , Li, C. , Wei, J. , & Kang, W. (2019). Antioxidant activity and total phenolic content of essential oils and extracts of sweet basil (*Ocimum basilicum* L.) plants. Food Science and Human Wellness, 8(3), 299–305.

[fsn34326-bib-0004] Auiewiriyanukul, W. , Saburi, W. , Kato, K. , Yao, M. , & Mori, H. (2018). Function and structure of GH 13_31 α‐glucosidase with high α‐(1→4) ‐glucosidic linkage specificity and transglucosylation activity. FEBS Letters, 592(13), 2268–2281. 29870070 10.1002/1873-3468.13126

[fsn34326-bib-0005] Bednarska, K. , Kuś, P. , & Fecka, I. (2020). Investigation of the phytochemical composition, antioxidant activity, and methylglyoxal trapping effect of *Galega officinalis* L. Herb in vitro. Molecules, 25(24), 5810. 33317096 10.3390/molecules25245810 PMC7764533

[fsn34326-bib-0006] Belkhiri, F. , Baghiani, A. , Zerroug, M. M. , & Arrar, L. (2017). Investigation of antihemolytic, xanthine oxidase inhibition, antioxidant and antimicrobial properties of *Salvia verbenaca* L. aerial part extracts. African Journal of Traditional, Complementary, and Alternative Medicines, 14(2), 273–281. 10.21010/ajtcam.v14i2.29 PMC5446453 28573244

[fsn34326-bib-0007] Boulfia, M. , Lamchouri, F. , Senhaji, S. , Lachkar, N. , Bouabid, K. , & Toufik, H. (2021). Mineral content, chemical analysis, in vitro antidiabetic and antioxidant activities, and antibacterial power of aqueous and organic extracts of *Moroccan Leopoldia comosa* (L.) parl. Bulbs. Evidence‐Based Complementary and Alternative Medicine, 2021, 1–17. 10.1155/2021/9932291 PMC8324349 34335845

[fsn34326-bib-0008] Buljeta, I. , Nosić, M. , Pichler, A. , Ivić, I. , Šimunović, J. , & Kopjar, M. (2022). Apple fibers as carriers of blackberry juice polyphenols: Development of natural functional food additives. Molecules, 27(9), 3029. 35566379 10.3390/molecules27093029 PMC9101031

[fsn34326-bib-0009] Chai, Y. , Wang, M. , & Zhang, G. (2013). Interaction between amylose and tea polyphenols modulates the postprandial glycemic response to high‐amylose maize starch. Journal of Agricultural and Food Chemistry, 61(36), 8608–8615. 23964645 10.1021/jf402821r

[fsn34326-bib-0010] Chukwuma, C. I. , Mashele, S. S. , & Akuru, E. A. (2020). Evaluation of the *in vitro* ⍺‐amylase inhibitory, antiglycation, and antioxidant properties of *Punica granatum* L. (pomegranate) fruit peel acetone extract and its effect on glucose uptake and oxidative stress in hepatocytes. Journal of Food Biochemistry, 44(5), e13175. 32160327 10.1111/jfbc.13175

[fsn34326-bib-0011] Ćorković, I. , Gašo‐Sokač, D. , Pichler, A. , Šimunović, J. , & Kopjar, M. (2022). Dietary polyphenols as natural inhibitors of α‐amylase and α‐glucosidase. Lifestyles, 12(11), 1692. 10.3390/life12111692 PMC9693262 36362847

[fsn34326-bib-0012] Dirar, A. I. , Alsaadi, D. H. M. , Wada, M. , Mohamed, M. A. , Watanabe, T. , & Devkota, H. P. (2019). Effects of extraction solvents on total phenolic and flavonoid contents and biological activities of extracts from Sudanese medicinal plants. South African Journal of Botany, 120, 261–267.

[fsn34326-bib-0013] Do, Q. D. , Angkawijaya, A. E. , Tran‐Nguyen, P. L. , Huynh, L. H. , Soetaredjo, F. E. , Ismadji, S. , & Ju, Y. H. (2014). Effect of extraction solvent on total phenol content, total flavonoid content, and antioxidant activity of *Limnophila aromatica* . Journal of Food and Drug Analysis, 22(3), 296–302. 28911418 10.1016/j.jfda.2013.11.001 PMC9354875

[fsn34326-bib-0014] Dorta, E. , Lobo, M. G. , & Gonzalez, M. (2012). Reutilization of mango byproducts: Study of the effect of extraction solvent and temperature on their antioxidant properties. Journal of Food Science, 77(1), C80–C88. 22132766 10.1111/j.1750-3841.2011.02477.x

[fsn34326-bib-0015] Familoni, O. B. , Asekun, O. T. , Okoh, O. , Asekunowo, A. K. , & Ashafa, A. O. (2019). Polyphenolic constituents, antioxidant and hypoglycemic potential of leaf extracts of *Acalypha godseffiana* from eastern Nigeria: *In vitro* study. Journal of Medicinal Plants for Economic Development, 3(1), 1–9.

[fsn34326-bib-0016] Farvin, K. S. , Surendraraj, A. , Al‐Ghunaim, A. , & Al‐Yamani, F. (2019). Chemical profile and antioxidant activities of 26 selected species of seaweeds from Kuwait coast. Journal of Applied Phycology, 31, 2653–2668.

[fsn34326-bib-0017] Fasakin, C. F. , Udenigwe, C. C. , & Aluko, R. E. (2011). Antioxidant properties of chlorophyll‐enriched and chlorophyll‐depleted polyphenolic fractions from leaves of *Vernonia amygdalina* and *Gongronema latifolium* . Food Research International, 44(8), 2435–2441.

[fsn34326-bib-0018] Fernandes, P. A. , Le Bourvellec, C. , Renard, C. M. , Nunes, F. M. , Bastos, R. , Coelho, E. , … Cardoso, S. M. (2019). Revisiting the chemistry of apple pomace polyphenols. Food Chemistry, 294, 9–18. 31126510 10.1016/j.foodchem.2019.05.006

[fsn34326-bib-0019] Fettach, S. , Mrabti, H. N. , Sayah, K. , Bouyahya, A. , Salhi, N. , Cherrah, Y. , & El Abbes, F. M. (2019). Phenolic content, acute toxicity of *Ajuga iva* extracts and assessment of their antioxidant and carbohydrate digestive enzyme inhibitory effects. South African Journal of Botany, 125, 381–385.

[fsn34326-bib-0020] Gong, T. , Yang, X. , Bai, F. , Li, D. , Zhao, T. , Zhang, J. , … Guo, Y. (2020). Young apple polyphenols as natural α‐glucosidase inhibitors: In vitro and in silico studies. Bioorganic Chemistry, 96, 103625. 32028059 10.1016/j.bioorg.2020.103625

[fsn34326-bib-0021] Goulas, V. , & Georgiou, E. (2019). Utilization of carob fruit as sources of phenolic compounds with antioxidant potential: Extraction optimization and application in food models. Food, 9(1), 20. 10.3390/foods9010020 PMC7022565 31878230

[fsn34326-bib-0022] Hachkova, H. , Nagalievska, M. , Soliljak, Z. , Kanyuka, O. , Kucharska, A. Z. , Sokół‐Łętowska, A. , & Sybirna, N. (2021). Medicinal plants, *Galega officinalis* L. and Yacon leaves as potential sources of antidiabetic drugs. Antioxidants, 10(9), 1362. 34572994 10.3390/antiox10091362 PMC8466348

[fsn34326-bib-0023] Hwang, P. A. , Hung, Y. L. , Tsai, Y. K. , Chien, S. Y. , & Kong, Z. L. (2015). The brown seaweed *Sargassum hemiphyllum* exhibits α‐amylase and α‐glucosidase inhibitory activity and enhances insulin release in vitro. Cytotechnology, 67, 653–660. 25344877 10.1007/s10616-014-9745-9 PMC4474997

[fsn34326-bib-0024] Irondi, E. A. , Akintunde, J. K. , Agboola, S. O. , Boligon, A. A. , & Athayde, M. L. (2017). Blanching influences the phenolics composition, antioxidant activity, and inhibitory effect of *Adansonia digitata* leaves extract on α‐amylase, α‐glucosidase, and aldose reductase. Food Science & Nutrition, 5(2), 233–242. 28265358 10.1002/fsn3.386 PMC5332274

[fsn34326-bib-0025] Jablonsky, M. , Majova, V. , Strizincova, P. , Sima, J. , & Jablonsky, J. (2020). Investigation of total phenolic content and antioxidant activities of spruce bark extracts isolated by deep eutectic solvents. Crystals, 10(5), 402.

[fsn34326-bib-0026] Jang, M. H. , Kim, H. Y. , Kang, K. S. , Yokozawa, T. , & Park, J. H. (2009). Hydroxyl radical scavenging activities of isoquinoline alkaloids isolated from *Coptis chinensis* . Archives of Pharmacal Research, 32, 341–345. 19387576 10.1007/s12272-009-1305-z

[fsn34326-bib-0027] Kadan, S. , Saad, B. , Sasson, Y. , & Zaid, H. (2016). *In vitro* evaluation of anti‐diabetic activity and cytotoxicity of chemically analyzed *Ocimum basilicum* extracts. Food Chemistry, 196, 1066–1074. 26593590 10.1016/j.foodchem.2015.10.044

[fsn34326-bib-0028] Karimi, A. , Azizi, M. H. , & Ahmadi Gavlighi, H. (2020). Fractionation of hydrolysate from corn germ protein by ultrafiltration: *In vitro* antidiabetic and antioxidant activity. Food Science & Nutrition, 8(5), 2395–2405. 32405396 10.1002/fsn3.1529 PMC7215226

[fsn34326-bib-0029] Karimi, A. , Gavlighi, H. A. , Sarteshnizi, R. A. , & Udenigwe, C. C. (2021). Effect of maize germ protein hydrolysate addition on digestion, *in vitro* antioxidant activity and quality characteristics of bread. Journal of Cereal Science, 97, 103148.

[fsn34326-bib-0030] Khezri, M. , Asghari Zakaria, R. , Zare, N. , & Johari‐Ahar, M. (2022). Improving galegine production in transformed hairy roots of *Galega officinalis* L. via elicitation. AMB Express, 12(1), 65. 35657528 10.1186/s13568-022-01409-7 PMC9166927

[fsn34326-bib-0031] Kolak, U. , Öztürk, M. , Özgökçe, F. , & Ulubelen, A. (2006). Norditerpene alkaloids from *delphinium linearilobum* and antioxidant activity. Phytochemistry, 67(19), 2170–2175. 16860354 10.1016/j.phytochem.2006.06.006

[fsn34326-bib-0032] Lalegani, S. , Gavlighi, H. A. , Azizi, M. H. , & Sarteshnizi, R. A. (2018). Inhibitory activity of phenolic‐rich pistachio green hull extract‐enriched pasta on key type 2 diabetes relevant enzymes and glycemic index. Food Research International, 105, 94–101. 29433292 10.1016/j.foodres.2017.11.003

[fsn34326-bib-0033] Lapornik, B. , Prošek, M. , & Wondra, A. G. (2005). Comparison of extracts prepared from plant by‐products using different solvents and extraction time. Journal of Food Engineering, 71(2), 214–222.

[fsn34326-bib-0034] Lim, S. , Choi, A. H. , Kwon, M. , Joung, E. J. , Shin, T. , Lee, S. G. , … Kim, H. R. (2019). Evaluation of antioxidant activities of various solvent extract from *Sargassum serratifolium* and its major antioxidant components. Food Chemistry, 278, 178–184. 30583359 10.1016/j.foodchem.2018.11.058

[fsn34326-bib-0035] Lohvina, H. , Sándor, M. , & Wink, M. (2021). Effect of ethanol solvents on Total phenolic content and antioxidant properties of seed extracts of fenugreek (*Trigonella foenum‐graecum* L.) varieties and determination of phenolic composition by HPLC‐ESI‐MS. Diversity, 14(1), 7.

[fsn34326-bib-0036] Ma, Y. L. , Sun, P. , Feng, J. , Yuan, J. , Wang, Y. , Shang, Y. F. , … Wei, Z. J. (2021). Solvent effect on phenolics and antioxidant activity of *Huangshan Gongju (Dendranthema morifolium* (Ramat) Tzvel. cv. Gongju) extract. Food and Chemical Toxicology, 147, 111875. 33227389 10.1016/j.fct.2020.111875

[fsn34326-bib-0037] Mamache, W. , Amira, S. , Ben Souici, C. , Laouer, H. , & Benchikh, F. (2020). In vitro antioxidant, anticholinesterases, anti‐α‐amylase, and anti‐α‐glucosidase effects of Algerian *Salvia aegyptiaca* and *Salvia verbenaca* . Journal of Food Biochemistry, 44(11), e13472. 33000487 10.1111/jfbc.13472

[fsn34326-bib-0038] Marinaccio, L. , Zengin, G. , Pieretti, S. , Minosi, P. , Szucs, E. , Benyhe, S. , … Mollica, A. (2023). Food‐inspired peptides from spinach rubisco endowed with antioxidant, antinociceptive and anti‐inflammatory properties. Food Chemistry: X, 18, 100640. 37008720 10.1016/j.fochx.2023.100640 PMC10064441

[fsn34326-bib-0039] McCalley, D. V. (2002). Analysis of the cinchona alkaloids by high‐performance liquid chromatography and other separation techniques. Journal of Chromatography A, 967(1), 1–19. 12219924 10.1016/s0021-9673(01)01557-6

[fsn34326-bib-0040] Nawaz, H. , Shad, M. A. , Rehman, N. , Andaleeb, H. , & Ullah, N. (2020). Effect of solvent polarity on extraction yield and antioxidant properties of phytochemicals from bean (*Phaseolus vulgaris*) seeds. *Brazilian* . Journal of Pharmaceutical Sciences, 56, e17129.

[fsn34326-bib-0041] Ngo, T. V. , Scarlett, C. J. , Bowyer, M. C. , Ngo, P. D. , & Vuong, Q. V. (2017). Impact of different extraction solvents on bioactive compounds and antioxidant capacity from the root of *Salacia chinensis* L. Journal of Food Quality, 2017, 1–8.

[fsn34326-bib-0042] Nijpels, G. , Boorsma, W. , Dekker, J. M. , Kostense, P. J. , Bouter, L. M. , & Heine, R. J. (2008). A study of the effects of acarbose on glucose metabolism in patients predisposed to developing diabetes: The Dutch acarbose intervention study in persons with impaired glucose tolerance (DAISI). Diabetes/Metabolism Research and Reviews, 24(8), 611–616. 18756586 10.1002/dmrr.839

[fsn34326-bib-0043] Noorolahi, Z. , Sahari, M. A. , Barzegar, M. , & Ahmadi Gavlighi, H. (2020). Tannin fraction of pistachio green hull extract with pancreatic lipase inhibitory and antioxidant activity. Journal of Food Biochemistry, 44(6), e13208. 32189358 10.1111/jfbc.13208

[fsn34326-bib-0044] Ogurtsova, K. , Guariguata, L. , Barengo, N. C. , Ruiz, P. L. D. , Sacre, J. W. , Karuranga, S. , … Magliano, D. J. (2022). IDF diabetes atlas: Global estimates of undiagnosed diabetes in adults for 2021. Diabetes Research and Clinical Practice, 183, 109118. 34883189 10.1016/j.diabres.2021.109118

[fsn34326-bib-0045] Pashazadeh, M. , Mirzazadeh, J. , & Alizadeh, A. (2015). Effect of Fabaceae (*Galega officinalis* L.) consumption on levels of blood glucose, lipids and lipoproteins in streptozotocin‐induced diabetic rats. Bulletin of Environment, Pharmacology and Life Sciences, 4(6), 127–132.

[fsn34326-bib-0046] Patel, S. B. , & Ghane, S. G. (2021). Phyto‐constituents profiling of *Luffa echinata* and *in vitro* assessment of antioxidant, anti‐diabetic, anticancer and anti‐acetylcholine esterase activities. Saudi Journal of Biological Sciences, 28(7), 3835–3846. 34220238 10.1016/j.sjbs.2021.03.050 PMC8241619

[fsn34326-bib-0047] Safitri, A. , Roosdiana, A. , Hitdatania, E. , & Damayanti, S. A. (2022). In vitro alpha‐amylase inhibitory activity of microencapsulated cosmos caudatus kunth extracts. Indonesian Journal of Chemistry, 22(1), 212–222.

[fsn34326-bib-0048] Sarteshnizi, R. A. , Sahari, M. A. , Gavlighi, H. A. , Regenstein, J. M. , Nikoo, M. , & Udenigwe, C. C. (2021). Influence of fish protein hydrolysate‐pistachio green hull extract interactions on antioxidant activity and inhibition of α‐glucosidase, α‐amylase, and DPP‐IV enzymes. LWT, 142, 111019.

[fsn34326-bib-0049] Seno, D. S. , Larasati, C. , Kamila, F. , Marwanto, Y. D. , Liwanda, N. , & Nurcholis, W. (2023). Effects of solvent combinations of Phenolics and antioxidants extraction from Kaempferia rotunda rhizomes. International Journal of Chemical and Biochemical Sciences, 23, 3.

[fsn34326-bib-0050] Shaheen, F. , Ahmad, M. , Khan, M. T. H. , Jalil, S. , Ejaz, A. , Sultankhodjaev, M. N. , … Choudhary, M. I. (2005). Alkaloids of *Aconitum laeve* and their anti‐inflammatory, antioxidant and tyrosinase inhibition activities. Phytochemistry, 66(8), 935–940. 15934134 10.1016/j.phytochem.2005.02.010

[fsn34326-bib-0051] Sinan, K. I. , Yagi, S. , Llorent‐Martínez, E. J. , Ruiz‐Medina, A. , Gordo‐Moreno, A. I. , Stefanucci, A. , … Zengin, G. (2023). Understanding the chemical composition and biological activities of different extracts of *Secamone afzelii* leaves: A potential source of bioactive compounds for the food industry. Molecules, 28(9), 3678. 37175088 10.3390/molecules28093678 PMC10180421

[fsn34326-bib-0052] Srivastava, R. , Mishra, N. , Tripathi, S. , & Mishra, N. (2020). Effect of solvents on antioxidant activities of *Feronia limonia* fruit. International Journal of Pharmaceutical Sciences and Research, 11(7), 3385–3391.

[fsn34326-bib-0053] Stefanucci, A. , Zengin, G. , Llorent‐Martinez, E. J. , Dimmito, M. P. , Della Valle, A. , Pieretti, S. , … Mollica, A. (2020). *Viscum album* L. homogenizer‐assisted and ultrasound‐assisted extracts as potential sources of bioactive compounds. Journal of Food Biochemistry, 44(9), e13377. 32713043 10.1111/jfbc.13377

[fsn34326-bib-0054] Sun, L. , Wang, Y. , & Miao, M. (2020). Inhibition of α‐amylase by polyphenolic compounds: Substrate digestion, binding interactions and nutritional intervention. Trends in Food Science & Technology, 104, 190–207.

[fsn34326-bib-0055] Sunagar, R. R. , & Sreerama, Y. N. (2023). Implication of solvent polarities on browntop millet (*Urochloa ramosa*) phenolic antioxidants and their ability to protect oxidative DNA damage and inhibit α‐amylase and α‐glucosidase enzymes. Food Chemistry, 411, 135474. 36681026 10.1016/j.foodchem.2023.135474

[fsn34326-bib-0056] Tierney, M. S. , Smyth, T. J. , Hayes, M. , Soler‐Vila, A. , Croft, A. K. , & Brunton, N. (2013). Influence of pressurised liquid extraction and solid–liquid extraction methods on the phenolic content and antioxidant activities of I rish macroalgae. International Journal of Food Science & Technology, 48(4), 860–869.

[fsn34326-bib-0057] Toma, A. , Makonnen, E. , Mekonnen, Y. , Debella, A. , & Addisakwattana, S. (2014). Intestinal α‐glucosidase and some pancreatic enzymes inhibitory effect of hydroalcholic extract of *Moringa stenopetala* leaves. BMC Complementary and Alternative Medicine, 14, 1–5. 24890563 10.1186/1472-6882-14-180 PMC4096440

[fsn34326-bib-0058] Tungmunnithum, D. , Thongboonyou, A. , Pholboon, A. , & Yangsabai, A. (2018). Flavonoids and other phenolic compounds from medicinal plants for pharmaceutical and medical aspects: An overview. Medicine, 5(3), 93. 10.3390/medicines5030093 PMC6165118 30149600

[fsn34326-bib-0059] Ullah, S. , Hussain, S. A. , Shaukat, F. , Hameed, A. , Yang, W. , & Song, Y. (2019). Antioxidant potential and the characterization of *Arachis hypogaea* roots. BioMed Research International, 1, 7073456. 10.1155/2019/7073456 PMC6948283 31950051

[fsn34326-bib-0060] Varghese, L. N. , & Mehrotra, N. (2020). α‐Amylase inhibitory activity of microencapsulated Nigella sativa L. and herb‐drug interaction: An in vitro analysis. Annals of Phytomedicine: An International Journal, 9(1), 107–112.

[fsn34326-bib-0061] Vergun, O. , Shymanska, O. , Rakhmetov, D. , Grygorieva, O. , Ivanišová, E. , & Brindza, J. (2020). Parameters of antioxidant activity of *Galega officinalis* L. and *Galega orientalis* lam. (Fabaceae Lindl.) plant raw material. Slovak Journal of Food Sciences, 14, 125–134.

[fsn34326-bib-0062] Wang, H. , Ye, Y. H. , Wang, H. H. , Liu, J. , Liu, Y. J. , & Jiang, B. W. (2019). HPLC‐QTOF‐MS/MS profiling, antioxidant, and α‐glucosidase inhibitory activities of *Pyracantha fortuneana* fruit extracts. Journal of Food Biochemistry, 43(5), e12821. 31353511 10.1111/jfbc.12821

[fsn34326-bib-0063] Wang, R. , Pan, F. , He, R. , Kuang, F. , Wang, L. , & Lin, X. (2021). Arecanut (*Areca catechu* L.) seed extracts extracted by conventional and eco‐friendly solvents: Relation between phytochemical compositions and biological activities by multivariate analysis. Journal of Applied Research on Medicinal and Aromatic Plants, 25, 100336.

[fsn34326-bib-0064] Wang, T. , Jonsdottir, R. , & Ólafsdóttir, G. (2009). Total phenolic compounds, radical scavenging and metal chelation of extracts from *Icelandic seaweeds* . Food Chemistry, 116(1), 240–248.

[fsn34326-bib-0065] Williamson, G. (2013). Possible effects of dietary polyphenols on sugar absorption and digestion. Molecular Nutrition & Food Research, 57(1), 48–57. 23180627 10.1002/mnfr.201200511

[fsn34326-bib-0066] Xie, X. , Chen, C. , & Fu, X. (2021). Screening α‐glucosidase inhibitors from four edible brown seaweed extracts by ultra‐filtration and molecular docking. LWT, 138, 110654.

[fsn34326-bib-0067] Yuliani, Y. (2022). Bioactivity effect of *Elephantopus scaber* Linn. Extracts against Spodoptera litura and the soil microbial community. Revista de Ciências Agroveterinárias, 21(3), 206–215.

[fsn34326-bib-0068] Zhang, H. , Wang, G. , & Dong, J. (2015). Inhibitory properties of aqueous ethanol extracts of propolis on alpha‐glucosidase. Evidence‐based Complementary and Alternative Medicine, 1, 587383. 10.1155/2015/587383 PMC4342172 25767553

[fsn34326-bib-0069] Zhang, L. , Xu, Q. , Li, L. , Lin, L. , Yu, J. , Zhu, J. , … Zang, H. (2020). Antioxidant and enzyme‐inhibitory activity of extracts from *Erigeron annuus* flower. Industrial Crops and Products, 148, 112283.

[fsn34326-bib-0070] Zhang, P. , Li, T. , Wu, X. , Nice, E. C. , Huang, C. , & Zhang, Y. (2020). Oxidative stress and diabetes: Antioxidative strategies. Frontiers of Medicine, 14, 583–600. 32248333 10.1007/s11684-019-0729-1

[fsn34326-bib-0071] Zhu, J. , Chen, C. , Zhang, B. , & Huang, Q. (2020). The inhibitory effects of flavonoids on α‐amylase and α‐glucosidase. Critical Reviews in Food Science and Nutrition, 60(4), 695–708. 30638035 10.1080/10408398.2018.1548428

